# A Flare-up of Systemic Lupus Erythematosus with Unusual Enteric Predominance

**DOI:** 10.7759/cureus.7068

**Published:** 2020-02-21

**Authors:** Joshua A Ronen, Armugam Mekala, Catherine Wiechmann, Sai Mungara

**Affiliations:** 1 Internal Medicine, Texas Tech University Health Sciences Center of the Permian Basin, Odessa, USA; 2 Internal Medicine, Texas Tech University Health Sciences Center, Lubbock, USA

**Keywords:** systemic lupus erythematosus (sle), enteritis, immunosuppressive treatment, pneumatosis intestinalis, submucosal vasculitis, intestinal pseudo-obstruction, surgical emergencies, protein-losing enteropathy

## Abstract

Enteritis associated with systemic lupus erythematosus (SLE) is a rare and unusual manifestation of the gastrointestinal (GI) consequences of SLE itself. Complications of the enteritis component include mesenteric vasculitis, intestinal pseudo-obstruction, and protein-losing enteropathy. Lupus enteritis is very responsive to treatment with pulse steroids in almost 70% of the patients, but it is critical to diagnose it early to prevent devastating organ damage.

The case describes a 21-year-old Caucasian female with a past medical history of uncomplicated laparoscopic appendectomy (one month prior to the time of presentation), major depressive disorder, asthma, iron deficiency anemia, pelvic inflammatory disease secondary to sexually transmitted Chlamydia trachomatis infection, and SLE (diagnosed two weeks prior to presentation). She had been transferred from an outside facility with complaints of severe right upper quadrant (RUQ) abdominal pain for one day. The patient had run out of her prescription for steroids and hydroxychloroquine two days prior to the presentation. Her abdominal pain was accompanied by nausea, bilious vomiting, non-bloody diarrhea, a photosensitive facial rash, left-sided pressure-type periorbital headache, diplopia, oral ulcers, inappetence, joint stiffness, and muscle weakness. A CT of the abdomen and pelvis from an outside facility showed enteritis involving the proximal jejunum with associated mesenteric edema and ascites, suggesting infectious versus inflammatory or autoimmune etiology. A repeat CT scan a few days later confirmed these findings along with adjacent mesenteric fat stranding. Her autoimmune workup confirmed the serological diagnosis of SLE, and assessment of the SLE Disease Activity Index (SLEDAI) confirmed the diagnosis of a severe SLE flare. Upper endoscopy detected edematous mucosa in the duodenum and jejunum without active bleeding, gastropathy, or ulceration. No surgical intervention was required. Her symptoms resolved with supportive care, pulse steroids, and hydroxychloroquine. She was discharged with instructions for outpatient follow-up with gastroenterology and rheumatology.

## Introduction

Systemic lupus erythematosus (SLE) is a well-studied autoimmune disorder with classification criteria set by the American College of Rheumatology (ACR). The malar or discoid rash, photosensitivity, and arthralgias are a few of the manifestations of this disease. Neurologic (i.e., seizures/psychosis), renal (i.e., nephritides), and hematologic insults (i.e., hemolytic anemia, leukopenia, thrombocytopenia) are also seen [1]. Less attention is given to SLE's enteric or gastrointestinal (GI) manifestations as such cases are few and far between. Protein-losing enteropathies, mesenteric vasculitides and vascular leakage, and intestinal pseudo-obstruction are observed in these manifestations [1]. Lupus enteritis (LE) is very responsive to treatment with pulse steroids. If unrecognized, however, LE may evolve into intestinal perforation and necrosis, thereby turning into a true surgical emergency [1]. Early recognition of the condition is critical to prevent this.

## Case presentation

**Figure 1:**
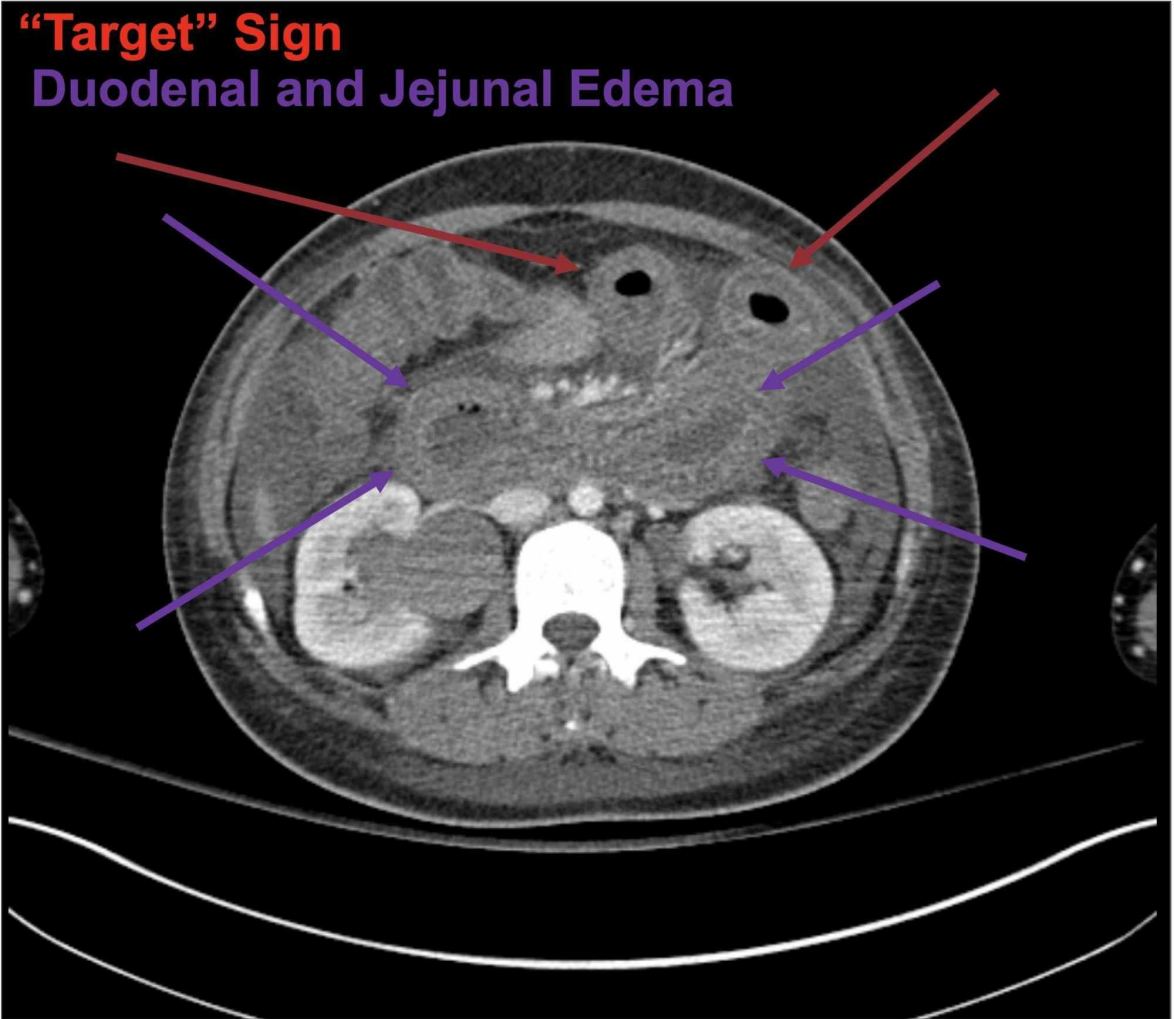
CT scan of the abdomen and pelvis with IV contrast Jejunum (purple arrows); duodenum (red arrows)

The patient was a 21-year-old Caucasian female with a past medical history of uncomplicated laparoscopic appendectomy (1 month prior to the time of presentation), major depressive disorder, asthma, iron deficiency anemia, pelvic inflammatory disease secondary to sexually transmitted Chlamydia trachomatis infection, and SLE. She presented to the emergency department from an outside hospital complaining of severe right upper quadrant abdominal (RUQ) pain for one day that was non-radiating. She had been hospitalized for two weeks prior to the time of this presentation with a de novo diagnosis of SLE with the debut of GI symptoms after running out of her original prescription for steroids and hydroxychloroquine two days prior. Overall, her abdominal pain was accompanied by fever, nausea, vomiting, diarrhea, headaches, diplopia, generalized muscle weakness, and arthralgias. She denied vaginal bleeding or discharge. Physical examination was most significant for diffuse tenderness to palpation of the abdomen especially in the RUQ, a negative Murphy's sign, and well-healing surgical incision sites. Vital signs remained within normal limits. An erythematous facial rash with a butterfly pattern was noted as well as tenderness to palpation of the metacarpophalangeal and interphalangeal joints of both hands. The differential diagnosis at this point included a severe lupus flare secondary to medication shortage, acute cholecystitis, acute pancreatitis, Celiac disease, or enterotoxigenic *Escherichia coli* (ETEC or traveler's diarrhea). Blood work including complete blood counts and metabolic profiles were negative for any acute processes during her admission except for an elevated lipase level three times above normal. A urine pregnancy test was also negative. Stool tests for white blood cells, gram staining, ova and parasites, and *Clostridium difficile *were also negative. A CT scan of the abdomen and pelvis with contrast showed moderate wall thickening of the duodenum and proximal jejunum and moderate adjacent-associated inflammatory stranding most compatible with infectious/inflammatory enteritis (Figure 1; obtained with consent from the hospital radiology department). Thus, we could safely rule out Fitz-Hugh-Curtis syndrome as well despite the patient's history of pelvic inflammatory disease.

**Figure 2:**
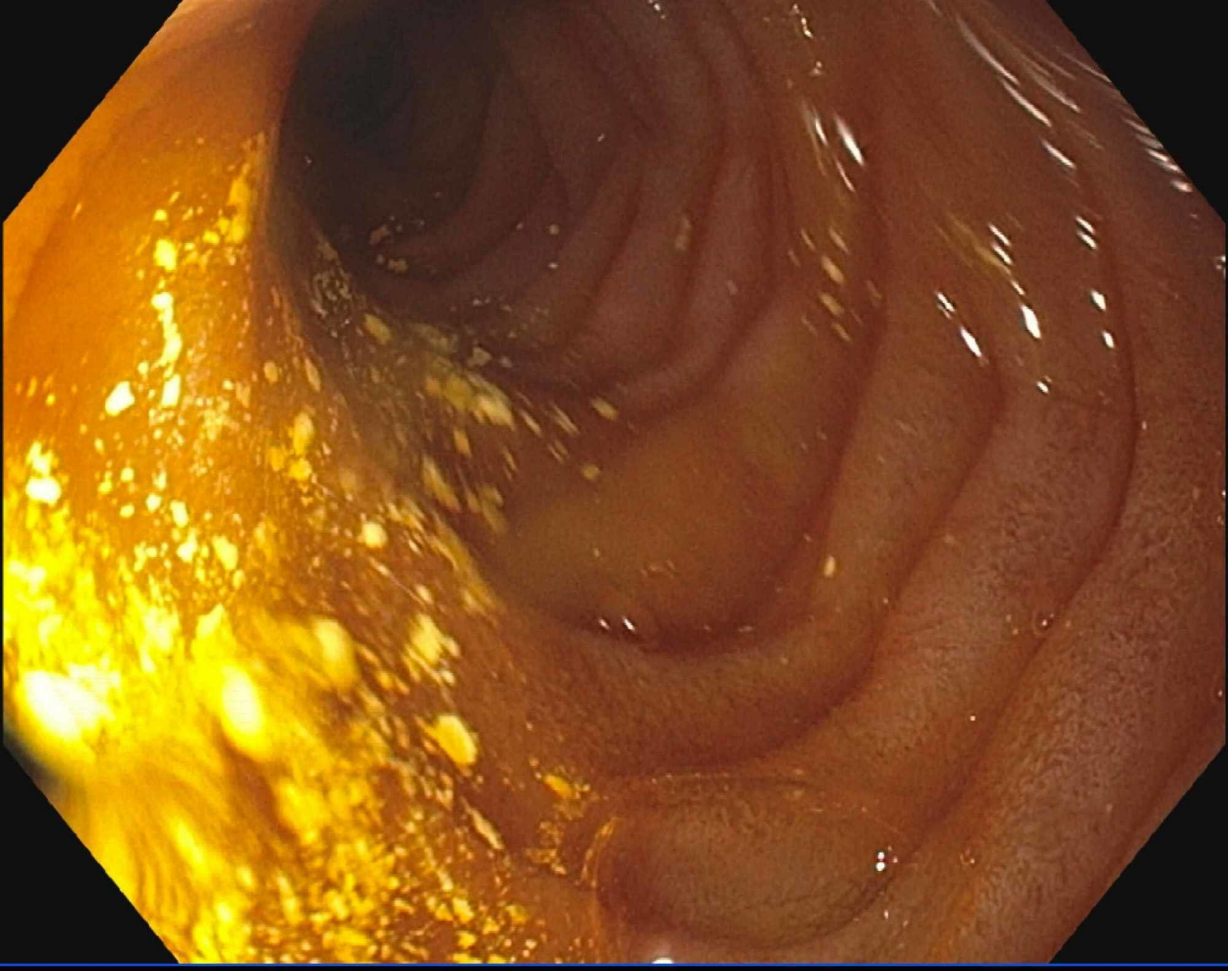
Findings from upper endoscopy showing edematous mucosa in the duodenum and jejunum

**Figure 3:**
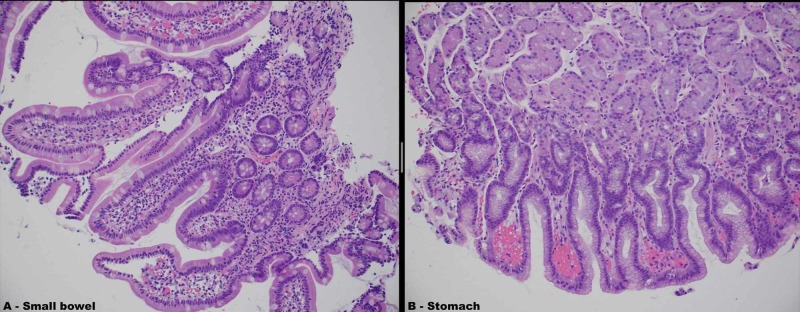
Stomach and small bowel biopsies obtained from upper endoscopy A) Pathology report: small bowel – lamina propria edema and vascular congestion; normal villous architecture; negative for increased intraepithelial lymphocytes, significant activity, and dysplasia B) Pathology report: stomach – gastric mucosa with reactive epithelial changes; immunohistochemical stain for *Helicobacter pylori* is negative; negative for significant activity and dysplasia

The case was determined to be non-surgical based on the lack of acute abdomen on exam and negative imaging findings. Gastroenterology planned for upper endoscopy (esophagogastroduodenoscopy, EGD). Rheumatology ordered autoimmunity standard tests and started the patient on pulse-dosed intravenous (IV) methylprednisolone (which was transitioned to oral prednisone after three days), oral hydroxychloroquine, and low-dose lisinopril once daily for scleroderma renal crisis (SRC) prophylaxis. EGD showed edematous mucosa in the duodenum and jejunum without active bleeding, gastropathy, or ulceration (Figure 2; obtained with consent from the hospital endoscopy department). Biopsies obtained from the EGD were non-specific (Figure 3, obtained with consent from the hospital pathology lab). The autoimmunity standard tests including anti-nuclear antibody (1:2560 titer), double-stranded DNA (1:80 titer), C3 (45 g/L), C4 (4 g/L), and anti-Smith (175 AU/mL) were strongly positive. Antiphospholipid antibodies were negative, as were anti-transglutaminase (TTG) antibodies for Celiac disease. This confirmed the diagnosis of a severe flare of lupus; more specifically, lupus enteritis secondary to medication shortage was determined to be the principal cause of the patient's abdominal pain. The patient continued to have intermittent bouts of moderate to severe periumbilical abdominal pains and non-bloody diarrhea that were treated with supportive care. Her symptoms responded very well to treatment by day four, and she had no further complications during her hospital stay.

## Discussion

Acute abdominal pain has a broad differential diagnosis. Brewer and Karmen suggest that GI symptoms can occur in approximately half of the patients with SLE, and they are triggered by infections or medication side effects [1]. Other clinical clues must often be applied to carefully differentiate between other comorbid GI conditions and lupus flares involving the stomach or small bowel. GI symptoms are most often nonspecific such as nausea, vomiting, weight loss, and abdominal pains. While abdominal pain is the most common presenting symptom of lupus, only 5.8% of patients with it are usually affected by its enteritis component; 85% of this subgroup tends to be females, with the condition usually identified almost three years after their initial diagnosis of SLE [1]. It is highly unlikely that LE will present on its own without other typical evidence of active lupus. The likelihood of a lupus patient presenting with an enteric component is directly proportional to how high their SLE Disease Activity Index (SLEDAI) is [1-2]. The differential remains wide for SLE patients with GI complaints, and only 10% of the patients are recognized clinically. Bert and Gertner emphasize that early recognition of LE is critical to prevent devastating organ damage and other life-threatening complications such as intestinal necrosis and perforation [3-4].

LE is commonly associated with complications such as mesenteric vasculitides and vascular leakage, intestinal pseudo-obstruction, and protein-losing enteropathy [1,5]. While our patient initially had an elevated lipase level three times the normal range and diffuse abdominal pain, we were not entirely convinced that she had pancreatitis based on her history of present illness. Her blood work and imaging results were not suggestive of any of these findings. Moreover, pancreatitis and hepatobiliary involvement are rarely encountered in LE patients [1]. While 15-55% of SLE patients present with transaminitis and other nonspecific GI symptoms, much of these can be secondary to the disease-modifying anti-rheumatic drugs (DMARDs) used to treat the condition itself (methotrexate, mycophenolate mofetil, corticosteroids, hydroxychloroquine, cyclophosphamide, cyclosporine, and azathioprine). However, Lee et al. have suggested that LE is the most common cause of acute abdominal pain in such patients [4]. 

**Figure 4:**
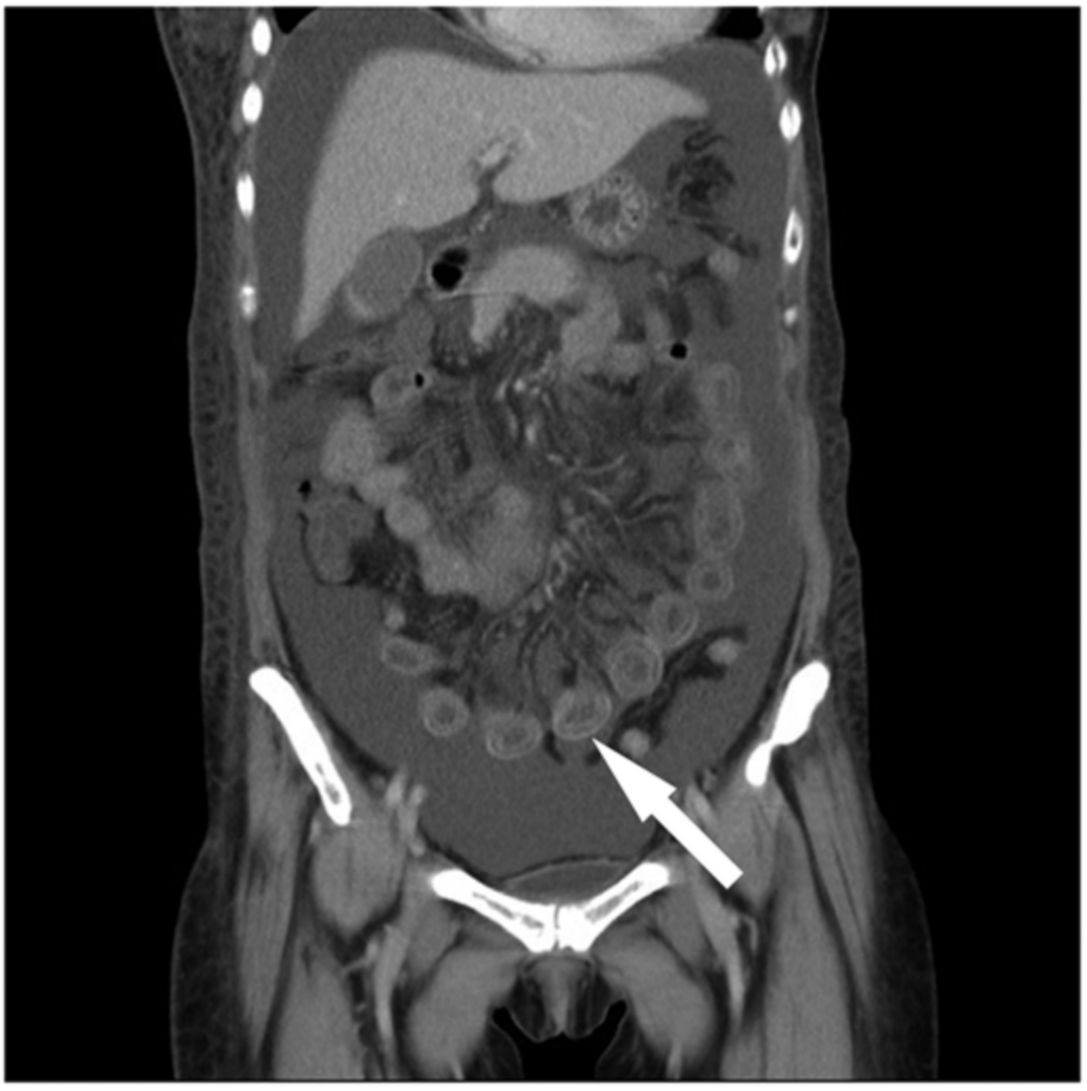
Target sign on CT scan of abdomen/pelvis in lupus enteritis Contrast-enhanced CT of the abdomen (coronal view) revealed target sign, which indicated diffuse circumferential wall thickening with submucosal edema of the entire small bowel, showing the ‘double halo’ or ‘target,’ sign (arrow)

According to Huang et al., characteristic findings of LE on CT scan of the abdomen and pelvis include bowel wall edema measuring >3 mm, abnormal bowel-wall enhancement and dilatation of the bowel lumen ("double halo" or "target" sign), and ascites as shown in Figure 1 and Figure 4 (Figure 4 was obtained with consent from QJM) [6-8]. Other imaging findings include the "Comb" sign (indicative of engorgement of the mesenteric vessels) and increased attenuation of the mesenteric fat [6]. Koo et al. conducted a retrospective analysis of SLE patients who experienced LE and underwent CT scans between January 1997 and December 2013; the symptoms in 74% of these patients with enteric complaints were compatible with CT findings [7]. The most common area of involvement of the GI tract is the jejunum (83%) and ileum (84%), although there are reports of pan-GI tract involvement as well [1]. Our patient's EGD, biopsy, and CT scan did demonstrate duodenal and jejunal edema. There have been ultrasound reports showing submucosal edematous thickening of the small intestine, making it useful for diagnosis when CT may be contraindicated [1]. According to Brewer and Kamen, in severe cases, the microscopic (histopathological) exam is consistent with the infiltration of the submucosal and muscular layer and necrotizing vasculitis with pan-mural predominant eosinophilic, neutrophilic, or mixed infiltrate. However, specific changes to suggest autoimmune disease or SLE alone are not typically seen in these biopsies and are not well-defined in pathologic literature.

Management

LE is a rare and poorly understood cause of abdominal pain in patients with lupus [8]. There is no standard treatment for it. However, based on the condition's responsiveness to high-dose pulse steroids, Janssens et al. have recommended them as the first-line treatment for LE [8]. For more severe flares or other organ involvement, cyclophosphamide or mycophenolate mofetil can be administered [8]. While recurrence rates are as high as 25% on steroids alone, there have been reports of successful treatment with no recurrence with immunomodulators (disease-modifying antirheumatic drugs, DMARDs) such as methotrexate, rituximab, and tacrolimus [1,8-10]. Furthermore, studies suggest that the presence of anti-phospholipid antibody syndrome (APS) could predict the rate of recurrence of enteritis in lupus patients. It has been shown that longer durations of high-dose IV corticosteroid therapy with methylprednisolone and eventual transition to oral prednisone therapy at 1 mg/kg/day can contribute greatly to the clinical and radiological resolution as well as the reduction in LE recurrence [11]. Clinicians should maintain a high index of suspicion for enteric complications in patients on steroid therapy aside from such of LE itself, although the medication-related side effects are very rare according to Alcocer-Gouyonnet et al [12]. 

## Conclusions

LE is a poorly recognized cause of abdominal pain in lupus patients, with distinct clinical and therapeutic features. The disease complications include mesenteric vasculitides, intestinal pseudo-obstruction, and protein-losing enteropathies. In very severe cases, it can be a surgical emergency secondary to intestinal necrosis and perforation. Currently, we have defined diagnostic strategies using laboratory studies and imaging modalities such as ultrasound and CT scan to help us narrow down our differentials for acute abdominal pain in lupus patients. High-dose corticosteroids have been heavily favored in existing literature as the primary treatment modality; they have also been reported to curb the likelihood of recurrence. However, every treatment can present side effects, either as a result of prolonged steroid therapy or the addition of other DMARDs. These should be closely monitored by primary care clinicians and sub-specialists such as Gastroenterologists and Rheumatologists. Overall, timely recognition and initiation of treatment for LE and its associated complications are critical for preventing significant morbidity and mortality in affected patients.
